# Induction of the growth arrest and DNA damage-inducible gene GADD153 by cisplatin in vitro and in vivo.

**DOI:** 10.1038/bjc.1994.455

**Published:** 1994-12

**Authors:** D. P. Gately, J. A. Jones, R. Christen, R. M. Barton, G. Los, S. B. Howell

**Affiliations:** Department of Biomedical Sciences, University of California, San Diego, La Jolla.

## Abstract

**Images:**


Br. J. Cancer (1994), 70, 1102–1106

© Macmillan Press Ltd., 1994

### Induction of the *g*rowth *a*rrest and *D*NA *d*amage-inducible gene *GADD153* by cisplatin *in vitro* and *in vivo*

D.P. Gately[1], J.A. Jones[2], R. Christen[2], R.M. Barton[2], G. Los[2] & S.B. Howell[1,2]

*Departments of [1]Biomedical Sciences and [2]Medicine, University of California, San Diego, 9500 Gilman Drive 0812, La Jolla, California, USA.*

**Summary** The inability to assess the extent of tumour damage immediately following treatment is a major clinical obstacle to improving the management of cancer patients. Normally, the effectiveness of chemotherapy or radiation therapy cannot be determined for at least several weeks after treatment. We studied the increase in mRNA of the *g*rowth *a*rrest and *D*NA *d*amage-inducible gene *GADD153* in human 2008 ovarian carcinoma cells *in vitro* and *in vivo* to determine whether treatment-induced increases in the level of *GADD153* mRNA could be used as a marker of the extent of tumour damage. *GADD153* mRNA was increased in a transient, dose-dependent manner by cisplatin (DDP) when the tumour cells were grown both *in vitro* and as tumour xenografts in nude mice. The magnitude of induction of *GADD153* mRNA did not vary significantly between different 2008 xenografts treated with equal doses of DDP, and *GADD153* mRNA induction correlated with the degree of *in vitro* cytotoxicity for two different schedules of drug exposure. DDP increased *GADD153* mRNA levels in melanoma and head and neck xenograft models as well. We conclude that the increase in *GADD153* mRNA can be used to detect tumour injury at time points as short as 24 h after administration of DDP.

Cisplatin (DDP) is one of the most effective chemotherapeutic agents in the treatment of ovarian, testicular and head and neck cancers. Unfortunately, these tumours characteristically develop resistance to DDP, and this contributes to treatment failure (Andrews & Howell, 1990; Timmer-Bosscha *et al.*, 1992). Early detection of intrinsic resistance could allow for treatment changes that would be beneficial to the patient, for example the immediate addition of radiation therapy to a chemotherapy programme. However, in most cases, it is impossible to detect the presence of resistance until weeks after the chemotherapeutic treatment when the tumour is found not to have decreased in size, or even continued to increase in volume.

Two recent developments hold out promise for the development of a molecular strategy potentially capable of providing information on the extent of tumour injury within 24 h of the initiation of treatment. First is the recent identification and cloning of a large number of DNA damage-inducible genes whose transcription is activated by cellular injury (reviewed by Holbrook & Fornace, 1991). Second is the development of polymerase chain reaction (PCR)-based methodology for accurately quantifying the level of expression of specific messages in very small numbers of cells (Horikoshi *et al.*, 1992; Los *et al.*, 1993*a*). Among the damage-inducible genes, *GADD153* is of particular interest as a candidate for early detection of tumour injury because it is induced to high levels by a large number of agents that cause either DNA damage or cell cycle arrest. These include UV light, hypoxia, serum starvation, medium depletion, cysteine conjugates, dithiothreitol and various chemotherapeutic drugs (Fornace *et al.*, 1989; Chen *et al.*, 1992; Luethy & Holbrook, 1992; Price & Calderwood, 1992). In addition, the induction is transient which, in principle, might permit repeated assessment of the magnitude of its induction with serial courses of treatment.

*GADD153* was originally cloned by hybridisation subtraction of mRNA from proliferating *vs* UV-treated CHO cells (Fornace *et al.*, 1988). It is one of a family of genes that is coordinately regulated by agents that induce growth arrest or DNA damage (Fornace *et al.*, 1989). *GADD153* is highly conserved in mammalian species; hamster *GADD153* shares 78% nucleotide sequence identity with the human gene (Park *et al.*, 1992) and >85% with the mouse gene (Ron & Habener, 1992). Although the function of *GADD153* in the damage response is unknown, it appears to be a modulator of the transcription factors C/EBP and LAP (Ron & Habener, 1992). Ron and Habener cloned CHOP-10, the mouse homologue of *GADD153*, by identifying proteins that could dimerise with CCAAT/enhancer-binding protein (C/EBP) or liver-enriched transcriptional activator protein (LAP), but not bind to the cytokine-responsive enhancer element APRE (acute-phase responsive element). They found that CHOP-10 localised in the nucleus and co-immunoprecipitated with LAP. They also found that overexpression of CHOP-10 inhibited the activation of an APRE-driven luciferase construct.

Recently, the same group has reported that *GADD153* is involved in the oncogenesis of human myxoid liposarcomas (Aman *et al.*, 1992; Crozat *et al.*, 1993). They demonstrated that the characteristic chromosomal translocation found in this tumour type creates a fusion protein of *GADD153* and a previously unreported RNA-binding protein (named TLS for *t*ranslocated in *l*ipo*s*arcoma; Crozat *et al.*, 1993). This fusion protein contains the DNA-binding and leucine zipper domains of the GADD153 protein fused to a domain in TLS that has a glycine-rich region similar to that of the transcription factor SP-1. They hypothesise that this translocation changes the effect of GADD153/CHOP-10 from a transcriptional suppressor to a oncogenic transcriptional activator.

We report here studies that demonstrate that the magnitude of the increase in *GADD153* messenger RNA can be used to detect and quantify the amount of tumour cell injury produced by DDP within 24 h of drug exposure both *in vitro* and *in vivo*, and that there is a good correlation between *GADD153* mRNA induction and the extent of cell kill *in vitro*.

### Materials and methods

### *Cell culture and conditions*

The human ovarian serous adenocarcinoma cell line 2008 (DiSaia *et al.*, 1972) was grown as a monolayer culture in a humidified incubator at 37°C and 5% carbon dioxide. 2008 cells were maintained in complete RPMI-1640 supplemented with 5% fetal calf serum and 2 mM glutamine. Cisplatin was obtained from Bristol laboratories (Princeton, NJ, USA).

Correspondence: D.P. Gately.
Received 19 May 1994; and in revised form 1 August 1994.

*Northern blotting*

Total cellular RNA was extracted by the guanidium isothiocyanate (GITC)–caesium chloride method (Davis *et al.*, 1986). The RNA was separated on 1% formaldehyde agarose gels and transferred to MagnaGraph nylon membranes (MSI, Westboro, MA, USA) by capillary transfer. The RNA was immobilised by baking at 80°C for 30 min. Probes were labelled with [$^{32}$P]dCTP using the Amersham Multiprime kit (Amersham, Arlington Heights, IL, USA). The blots were prehybridised, hybridised and washed as described in Maniatis *et al.* (Sambrook *et al.*, 1989). The blots were exposed to film (Fuji, RX-50 X-ray film) and quantitated either by laser densitometry (LKB Ultroscan XL, Bromma, Sweden) or by the Molecular Imager System (Bio-Rad, Hercules, CA, USA). The human *GADD153* probe was a gift from Dr N.J. Holbrook (NIA, NIH, Baltimore, MD, USA). Lane loading differences were corrected for by comparison to the same blot hybridised with a β-actin probe.

In vivo *experiments*

Five million 2008 cells were injected subcutaneously above the foreshoulder of female *nu/nu* mice (Harlan, Sprague-Dawley, Indianopolis, IL, USA). The xenografts were allowed to grow to a volume of approximate 1 ml. At that time, the mice were treated intraperitoneally with DDP. The mice were sacrificed and the tumours were removed at the appropriate time point. The tumours were homogenised using a Polytron homogenizer (Biospec Products, Bartlesville, OK, USA) in GITC buffer. RNA was extracted as described above.

### Results

*Dose dependence of the increase in* GADD153 *mRNA levels after DDP treatment* in vitro

In order to determine whether DDP increased *GADD153* mRNA levels in exponentially growing human ovarian carcinoma 2008 cells, cultures were treated with 5, 10, 20 or 30 μM DDP for 1 h and then returned to drug-free medium. RNA extracted at 24 h after DDP treatment was subjected to Northern analysis. Figure 1 shows that *GADD153* mRNA expression was increased by DDP treatment, and that this increase was approximately linear with DDP concentration up to 30 μM. A representative blot is shown in Figure 1a; the quantitation of the autoradiogram is shown graphically in Figure 1b.

*Time course of the increase in* GADD153 *mRNA levels after DDP treatment* in vitro

Figure 2 shows the time course of the increase in *GADD153* mRNA levels after DDP treatment in 2008 cells growing *in vitro*. The cells were treated with 20 μM DDP for 1 h and RNA was extracted at 8, 24, 48, 96 and 120 h after DDP exposure. An increase in *GADD153* mRNA level was evident at 8 h; this increase had peaked at 24 h, and had returned to basal levels by 120 h when the cells had returned to exponential growth.

*Effect of DDP exposure schedule on* GADD153 *mRNA levels* in vitro

In order to determine whether the increase in *GADD153* mRNA caused by DDP was dependent on the schedule of drug exposure, *GADD153* mRNA levels were measured in cells treated with equitoxic exposures using two different DDP regimens. In the 2008 ovarian carcinoma cell line the $IC_{50}$ for a 24 h exposure to DDP is 10-fold less than the $IC_{50}$ for a 1 h exposure (data not shown). A 1 h treatment with 5 μM DDP and a 24 h treatment with 0.5 μM DDP both resulted in inhibition of colony formation by 50%. As shown in Figure 3, such equitoxic doses of DDP resulted in approximately equal increases of *GADD153* mRNA measured at 24 h after the start of drug exposure. This indicates that, over the range studied, the schedule of drug exposure had little effect on the magnitude of the injury response.

*Dose dependence of the increase in* GADD153 *mRNA levels after DDP treatment* in vivo

The ability of DDP to increase *GADD153* mRNA levels *in vivo* was investigated in 2008 xenografts growing subcutaneously in nude mice. Implanted cells were allowed to

**Figure 1** The effect of DDP on *GADD153* message levels. Exponentially growing 2008 cells were treated with DDP for 1 h and returned to drug-free medium for 24 h. *GADD153* mRNA induction was assayed by Northern blot analysis. **a**, A representative Northern blot. **b**, The hybridisation has been quantitated and is shown graphically. Lane loading is corrected by ratio to β-actin and values are relative to untreated control cells. Error bars ± s.e.m.

**Figure 2** The effect of post-treatment time on *GADD153* message levels after a 1 h exposure to 20 μM DDP. Exponentially growing 2008 cells were treated with 20 μM DDP for 1 h and returned to drug-free medium for 8, 24, 48, 96 and 120 h. *GADD153* mRNA levels were assayed by Northern blot analysis. Values are corrected for β-actin and reported relative to untreated control cells. Error bars ± s.e.m.

grow into a tumour with a volume of approximately 1 ml, and then the mice were treated with 15, 30, 50 or 100 mg $kg^{-1}$ DDP administered as a single intraperitoneal injection. Figure 4 shows the results obtained when RNA was harvested at 24 h after drug treatment and subjected to Northern analysis. DDP increased *GADD153* mRNA in the 2008 xenografts in a dose-dependent manner. The smallest dose that produced a reproducibly measurable increase in *GADD153* message was 15 mg $kg^{-1}$. At a dose of 100 mg $kg^{-1}$, the fold induction of *GADD153* message reached $7.2 \pm 0.5$ (s.e.m.).

*Time course of the increase in* GADD153 *mRNA levels after DDP treatment* in vivo

The time course of the increase in *GADD153* mRNA was investigated in 2008 xenografts in nude mice following administration of DDP at a dose of 30 mg $kg^{-1}$ by the intraperitoneal route. Increased levels of *GADD153* mRNA were detectable at 24 h, were maximal at 48 h and had returned toward baseline by 72 h following DDP treatment (Figure 5). Therefore, as was found *in vitro*, the increase in *GADD153* mRNA levels was transient *in vivo* and levels returned toward baseline sufficiently rapidly that one would expect the DDP-induced transcriptional activation of *GADD153* to have resolved prior to the next dose of chemotherapy even if given on a weekly schedule.

*Heterogeneity of* GADD153 *mRNA levels after DDP treatment* in vivo

In order to use the magnitude of increase in *GADD153* mRNA as a surrogate for clinical tumour regression, the coefficient of variation between biopsies must be relatively small. Variations in the increase of *GADD153* mRNA levels produced by equal doses of DDP in separate tumours were studied in the *in vivo* model. Northern analysis of 2008 xenografts using RNA harvested 24 h following a single intraperitoneal dose of DDP 30 mg $kg^{-1}$ demonstrated a 3.2-, 4.0-, 3.9- and 3.6-fold increase in *GADD153* mRNA in four tumours, which yielded a mean ± s.d. of $3.7 \pm 0.4$ and a coefficient of variation of 11.7%.

*Increase in* GADD153 *mRNA levels produced by DDP treatment in melanoma and head and neck cancer xenografts*

The ability of DDP to increase *GADD153* mRNA levels *in vivo* was investigated in two additional xenografts, T289 human melanoma and UMSCC 10b human squamous cell carcinoma, growing subcutaneously in nude mice. Implanted cells were allowed to grow into a tumour with a volume of approximately 1 ml, and then mice were treated with 50 mg $kg^{-1}$ DDP administered as a single intraperitoneal injection. RNA from the tumours was extracted 24 h later and analysed by Northern blot. Table 1 shows that DDP increases the levels of *GADD153* mRNA in a variety of human tumour xenografts of different types.

**Figure 3** The effect of equitoxic DDP treatments on the induction of *GADD153* mRNA. Exponentially growing 2008 cells were treated with $IC_{50}$, $IC_{90}$, or $IC_{99}$ exposures of DDP using either a 1 h (■) or a 24 h (●) duration of treatment. *GADD153* mRNA levels were assayed by Northern blot analysis 24 h after the start of DDP exposure. Values are corrected for β-actin and reported relative to untreated control cells. Error bars ± s.e.m.

**Figure 4** The effect of intraperitoneal DDP on *GADD153* message levels in 2008 xenografts in nude mice. Mice bearing 2008 xenografts were treated with DDP, RNA was harvested 24 h later and *GADD153* mRNA levels were assayed by Northern blot analysis. Values are corrected for β-actin and are reported relative to tumours from untreated animals. Error bars ± s.e.m.

### Discussion

A large number of genes that are induced by DNA damage have now been identified. We hypothesised that the magnitude of induction of one or more of these could provide a

**Figure 5** The effect of post-treatment time on *GADD153* message levels in 2008 xenografts in nude mice. Mice bearing 2008 xenografts were treated with 30 mg $kg^{-1}$ DDP, and RNA was harvested 24, 48 or 72 h later. *GADD153* mRNA levels were assayed by Northern blot analysis. Values are corrected for β-actin and are reported relative to tumours from untreated animals. Error bars ± s.e.m.

**Table I** Increase in *GADD153* mRNA in human tumour xenografts following 50 mg $kg^{-1}$ DDP

| *Cell line* | *Tumour type* | *Fold* GADD153 *mRNA increase ± s.e.m.* |
|---|---|---|
| 2008 | Ovarian carcinoma | 5.1 ± 1.3 |
| T289 | Melanoma | 8.1 ± 0.6 |
| UMSCC 10b | Squamous cell carcinoma | 3.0 ± 0.4 |

molecular approach to the long-standing clinical problem of how to rapidly determine whether a tumour is destined to respond to treatment that has recently been administered. Among the *gadd* family of genes, *GADD153* has a number of traits that make it a strong candidate for this role of surrogate marker. First, *GADD153* has low basal expression such that even relatively small degrees of induction can be detected. Second, unlike many of the other damage-inducible genes, it is not highly induced by treatment of the cells with phorbol ester tumour promoters (Holbrook & Fornace, 1991), and, by inference, may be less susceptible to induction by environmental factors. Third, *GADD153* is not cell cycle regulated. If tumour injury was monitored with a gene whose transcription was activated by both injury and an increase in growth rate, such as c-*fos* or c-*jun*, then it might be difficult to distinguish between impending response and the increase in the growth fraction that often accompanies partially successful chemotherapy. Finally, the increase in *GADD153* mRNA is an event that is quite far downstream in the sequence of steps that lead from administration of chemotherapy to tumour cell death. The magnitude of this increase reflects the net effect of several factors, including the amount of drug given to the patient, the total plasma exposure, how much of the drug actually got into the tumour cell, how much found its critical target within the cell, and how effectively cellular defence mechanisms offset the action of the drug. Thus, in principle, it reflects the extent to which the tumour has been injured. One can reasonably expect that this will be much more tightly linked to clinical response than post-treatment measurements such as plasma drug concentration or even total tumour drug level.

The expression of a number of genes, including *HSP60* (Kimura *et al.*, 1993) and *ERCC1* (Dabholkar *et al.*, 1992), has been postulated to be predictive of how well a tumour will respond to DDP. The pretreatment measurement of these genes can detect intrinsic resistance or differences in DNA repair capability. Since *GADD153* is measured after DDP treatment, the increase in mRNA levels reflects tumour damage and may give different information from pretreatment measurement of *HSP60* or *ERCC1*. At present a comparison of the correlation between the levels of these genes and the clinical outcome of the treatment has not been undertaken. It is important to point out that the level of *GADD153* mRNA expression at 24 h post treatment is likely to be a complex function of both the amount of damage done to DNA and the ability of the cell to repair this damage.

In approaching the use of *GADD153* as a potential molecular marker of injury, we chose to focus on a single widely used chemotherapeutic agent, DDP, and a type of tumour, ovarian carcinoma, that initially responds well to DDP treatment but for which acquired resistance usually becomes a major clinical problem (Andrews *et al.*, 1990; Perez *et al.*, 1991). Using the human ovarian carcinoma cell line 2008, the results indicate that *GADD153* can be induced 12-fold by levels of drug that result in approximately 10% survival of the original population of cells. The magnitude of the induction was predictably dependent on the extent of tumour cell kill, and it was relatively independent of the schedule of drug administration (i.e. 1 *vs* 24 h exposure). In addition, we confirmed that the time course of induction and return of mRNA levels to their basal level was such that it would be appropriate to use *GADD153* induction as a measure of injury on serial courses of treatment spaced more than a week or so apart. The time course results that we obtained with the human 2008 cells exposed to DDP differ considerably from the induction of *GADD153* by methyl methanesulphonate (MMS) in CHO cells. In the latter system Fornace *et al.* (1989) reported that induction was maximal at the end of a 4 h exposure to drug. At the present time no other explanations for these differences are available save for the fundamental differences in cellular system and drug tested. For example, MMS forms monoadducts with DNA (Friedberg, 1985), DDP forms both mono- and biadducts with DNA (Pinto & Lippard, 1985). These different types of adducts may have different detection and repair systems that could account for the differences in induction time.

Importantly, the results obtained in this study indicate that *GADD153* mRNA was increased *in vivo* in xenografts of several different types by doses of DDP that are clinically relevant, and that the time course of induction parallels that observed *in vitro*. In patients, DDP is commonly administered to cancer patients at doses of 90–100 mg $m^{-2}$, and some clinical trials are experimenting with doses as high as 150 mg $m^{-2}$ (Los *et al.*, 1993*b*). Los *et al.* (1994) have shown that in patients with head and neck tumours who show a complete response to DDP, *GADD153* mRNA levels have increased an average of 3.25-fold. Although the LD10 for DDP in nude mice is 15 mg $kg^{-1}$ (unpublished data), using the empirically derived conversion formulas of Freireich *et al.* (1966), a DDP dose of 30 mg $kg^{-1}$ in the mouse can be expected to generate the same degree of toxicity as a dose of 92 mg $m^{-2}$ in a human. Thus, our data suggest that the magnitude of induction of *GADD153* by doses commonly used in the clinic will be sufficient to permit its use as a measure of the extent to which a tumour believes it has been injured 24 h after initiation of therapy.

The ability to increase *GADD153* mRNA to high levels by doses of drug that are sufficient to cause tumour regression is only the first step in developing a strategy for rapid assessment of tumour injury. Given the heterogeneous nature of many tumours, how reliable would any one measurement of *GADD153* expression be in a single biopsy from a given tumour? Comparing a small number of 2008 xenografts, we found a coefficient of variation of only 11.7%, suggesting that within this type of tumour, measurement of *GADD153* mRNA may be associated with an acceptably small variance. However, *GADD153* mRNA levels from multiple biopsies of single tumours will have to be extensively studied in order to ensure that the variation is small in heterogeneous tumour cell populations from patients.

The prospect of translating the use of *GADD153* induction into a clinically useful tool has been much improved by development of PCR-based technologies that permit quantitative measurement of mRNA levels in as few as 1,000 cells (Los *et al.*, 1993*a*). Sufficient cellular material can be obtained from a single fine needle aspiration biopsy to permit accurate quantification of *GADD153* mRNA level before and again 24 h after treatment (Los *et al.*, 1994). Additional studies are needed to determine the variance associated with measurements made on needle biopsies obtained from different sections of the same tumour as well as from the same tumour before and after treatment. Also, further validation of the assay will be needed to confirm that there is a good correlation between the magnitude of *GADD153* induction and the clinical response of the tumour. It is important to point out that changes in the magnitude of induction of *GADD153* during serial courses of the same therapy may provide a method for detection of drug resistance at a time well before it becomes clinically apparent.

In this study we focused on *GADD153* induction by the chemotherapeutic agent DDP. However, since *GADD153* can be induced by a large number of chemotherapeutic agents as well as ultraviolet radiation (Luethy & Holbrook, 1992; D.P. Gately, unpublished data), it may be possible to use the induction of this gene to monitor the extent of tumour injury produced by many modalities of treatment.

**Abbreviations**: DDP, cisplatin; gadd, growth arrest and DNA damage; $IC_{50}$, concentration of drug required to inhibit colony formation by 50%; GITC, guanidine isothiocyanate; CHO, Chinese hamster ovary; C/EBP, CCAAT/enhancer-binding protein; LAP, liver-enriched transcriptional activator protein; APRE, acute-phase response element.

This work was supported in part by Grant DHP-26E from the American Cancer Society and Grant 100R47 from Bristol-Myers Squibb. This work was conducted in part by the Clayton Foundation for Research – California Division. Drs Howell, Los and Christen are Clayton Foundation Investigators. Contributions to this work by D.P. Gately are in partial fulfilment of the PhD requirements in the Department of Biomedical Sciences. Portions of this work were presented at the Annual Meetings of the American Association for Cancer Research and the American Society for Clinical Oncology, 1993.

### References

AMAN, P., RON, D., MANDAHL, N., FIORETOS, T., HEIM, S., ARHEDEN, K., WILLEN, H., RYDHOLM, A. & MITELMAN, F. (1992). Rearrangement of the transcription factor gene *CHOP* in myxoid liposarcomas with t(12;16)(q13;pp11). *Genes Chrom. Cancer*, **5**, 278-285.

ANDREWS, P.A. & HOWELL, S.B. (1990). Cellular pharmacology of cisplatin: perspectives on mechanisms of acquired resistance. *Cancer Cells*, **2**, 35-43.

ANDREWS, P.A., JONES, J.A., VARKI, N.M. & HOWELL, S.B. (1990). Rapid emergence of acquired *cis*-diamminedichloroplatinum(II) resistance in an *in vivo* model of human ovarian carcinoma. *Cancer Commun.*, **2**, 93-100.

CHEN, Q., YU, K., HOLBROOK, N.J. & STEVENS, J.L. (1992). Activation of the growth arrest and DNA damage-inducible gene *gadd 153* by nephrotoxic cysteine conjugates and dithiothreitol. *J. Biol. Chem.*, **267**, 8207-8212.

CROZAT, A., AMAN, P. & RON, D. (1993). Fusion of CHOP to a novel RNA-binding protein in human myxoid liposarcoma. *Nature*, **363**, 640-644.

DABHOLKAR, M., BOSTICK-BRUNTON, F., WEBER, C., BOHR, V.A., EGWUAGU, C. & REED, E. (1992) ERCC1 and ERCC2 expression in malignant tissues from ovarian cancer patients. *J. Natl Cancer Inst.*, **84**, 1512-1517.

DAVIS, L.G., DIBNER, M.D. & BATTEY, J.F. (1986). *Basic Methods in Molecular Biology*. Elsevier: New York.

DISAIA, P.J., SINKOVICS, J.G., RUTLEDGE, F.N. & SMITH, J.P. (1972). Cell-mediated immunity to human malignant cells. *Am. J. Obstet. Gynecol.*, **114**, 979-989.

FORNACE, A.J., ALAMO, I. & HOLLANDER, M.C. (1988). DNA damage-inducible transcripts in mammalian cells. *Proc. Natl Acad. Sci. USA*, **85**, 8800-8804.

FORNACE, A.J., NEBERT, D.W., HOLLANDER, C., LUETHY, J.D., PAPATHANASIOU, M., FARGNOLI, J. & HOLBROOK, N. (1989). Mammalian genes coordinately regulated by growth arrest signal and DNA damaging agents. *Mol. Cell. Biol.*, **9**, 4196-4203.

FREIREICH, E.J., GEHAN, E.A., RALL, D.P., SCHMIDT, L.H. & SKIPPER, H.E. (1966). Quantitative comparison of toxicity of anticancer agents in mouse, rat, hamster, dog, monkey and man. *Cancer Chemother. Rep.*, **50**, 219-244.

FRIEDBERG, E.C. (1985). *DNA Repair*. W.H. Freeman: New York.

HOLBROOK, N.J. & FORNACE, A.J. (1991). Response to adversity: molecular control of gene activation following genotoxic stress. *New Biol.*, **3**, 825-833.

HORIKOSHI, T., DANENBERG, K.D., STADLBAUER, T.H.W., VOLKENANDT, M., SHEA, L.C., AIGNER, K., GUSTAVSSON, B., LEICHMAN, L., FRÖSING, R., RAY, M., GIBSON, N.W., SPEARS, C.P. & DANENBERG, P.V. (1992). Quantitation of thymidylate synthase, dihydrofolate reductase a DT-diaphorase gene expression in human tumors using polymerase chain reaction. *Cancer Res.*, **52**, 108-116.

KIMURA, E., ENNS, R.E., ALCARAZ, J.E., ARBOLEDA, J., SLAMON, D.J. & HOWELL, S.B. (1993). Correlation of the survival of ovarian cancer patients with mRNA expression of the 60-kD heat-shock protein HSP-60. *J. Clin. Oncol.*, **11**, 891-898.

LOS, G., BARTON, B., VAN VEELEN, L., WOODS, A., VICARIO, D., ROBBINS, K.T. & HOWELL, S.B. (1994). Association between DNA damage-inducible gene expression in tumor response. *Proc. Am. Assoc. Cancer Res.*, **35**, 3277.

LOS, G., BARTON, R.M., NAKATA, B., LEE, L.K., VAN VEELEN, L.R. & HOWELL, S.B. (1993*a*). DNA damage early-response genes as a tool to monitor tumor injury following chemotherapy. *Proc. Am. Assoc. Cancer Res.*, **35**, 2582.

LOS, G., ROBBINS, T.K., BARTON, R.M., HANCHETT, C.A., HEATH, D.D., VICARIO, D. & HOWELL, S.B. (1993*b*). Quantitation of tumor platinum content in patients receiving superselective arterial infusion (S.A.I.) of high dose cisplatin (cDDP) in advanced head and neck cancers: a preliminary report. *Proc. Am. Soc. Clin. Oncol.*, **12**, 919.

LUETHY, J.D. & HOLBROOK, N.J. (1992). Activation of the *gadd 153* promoter by genotoxic agents: a rapid and specific response to DNA damage. *Cancer Res.*, **52**, 5-10.

PARK, J.S., LUETHY, J.D., WANG, M.G., FARGNOLI, J., FORNACE, A.J., MCBRIDE, O.W. & HOLBROOK, N.J. (1992). Isolation, characterization and chromosomal localization of the human *GADD153* gene. *Gene*, **116**, 259-267.

PEREZ, R.P., GODWIN, A.K., HAMILTON, T.C. & OZOLS, R.F. (1991). Ovarian cancer biology. *Semin. Oncol.*, **18**, 186-204.

PINTO, A.L. & LIPPARD, S.J. (1985). Binding of the antitumor drug *cis*-diamminedichloroplatinum(II) (cisplatin) to DNA. *Biochim. Biophys. Acta*, **780**, 167-180.

PRICE, B.D. & CALDERWOOD, S.K. (1992). Gadd45 and Gadd153 messenger RNA levels are increased during hypoxia and exposure of cells to agents which elevate the levels of glucose-related proteins. *Cancer Res.*, **52**, 3814-3817.

RON, D. & HABENER, J.F. (1992). CHOP, a novel developmentally regulated nuclear protein that dimerizes with transcription factors C/EBP and LAP and functions as a dominant negative inhibitor of gene transcription. *Genes Dev.*, **6**, 439-453.

SAMBROOK, J., FRITSCH, E.F. & MANIATIS, T. (1989). *Molecular Cloning: A Laboratory Manual*. Cold Spring Harbor Laboratory Press: Cold Spring Harbor, NY.

TIMMER-BOSSCHA, H., MULDER, N.H. & DE VRIES, E.G.E. (1992). Modulation of *cis*-diamminedichloroplatinum(II) resistance: a review. *Br. J. Cancer*, **66**, 227-238.


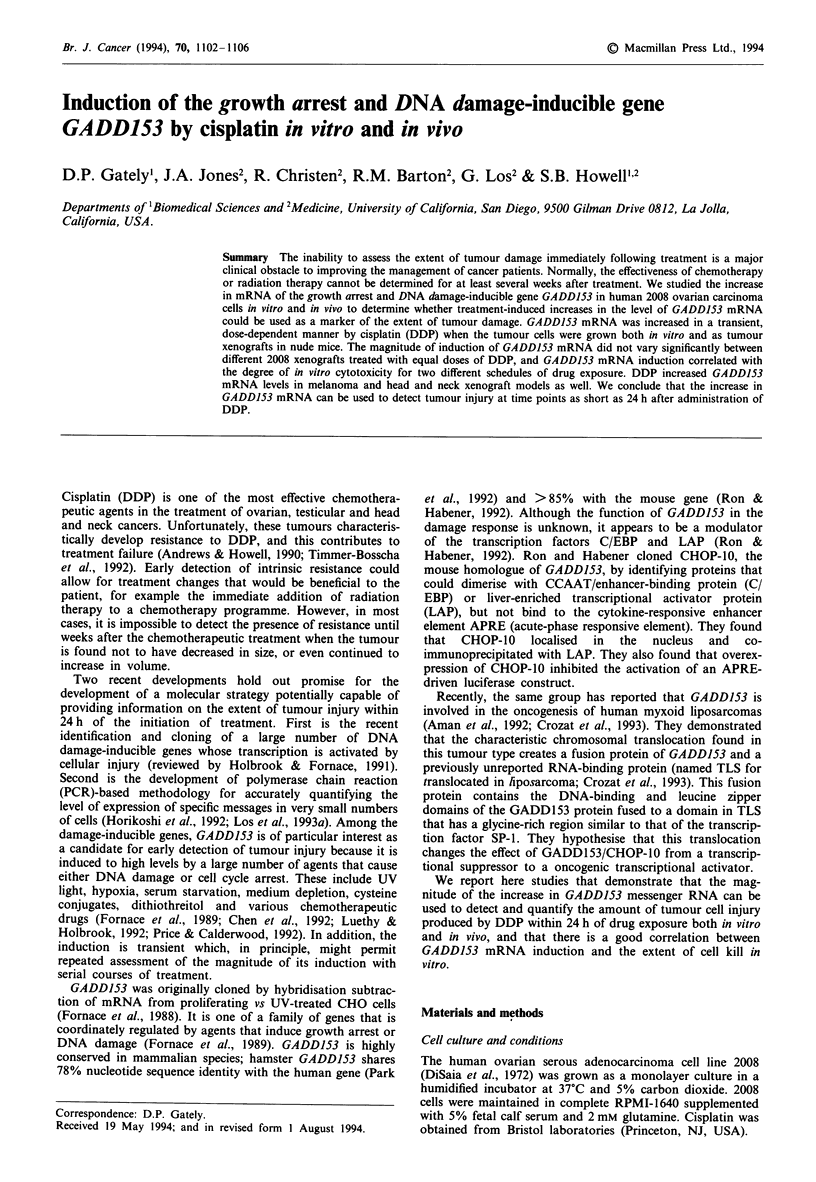

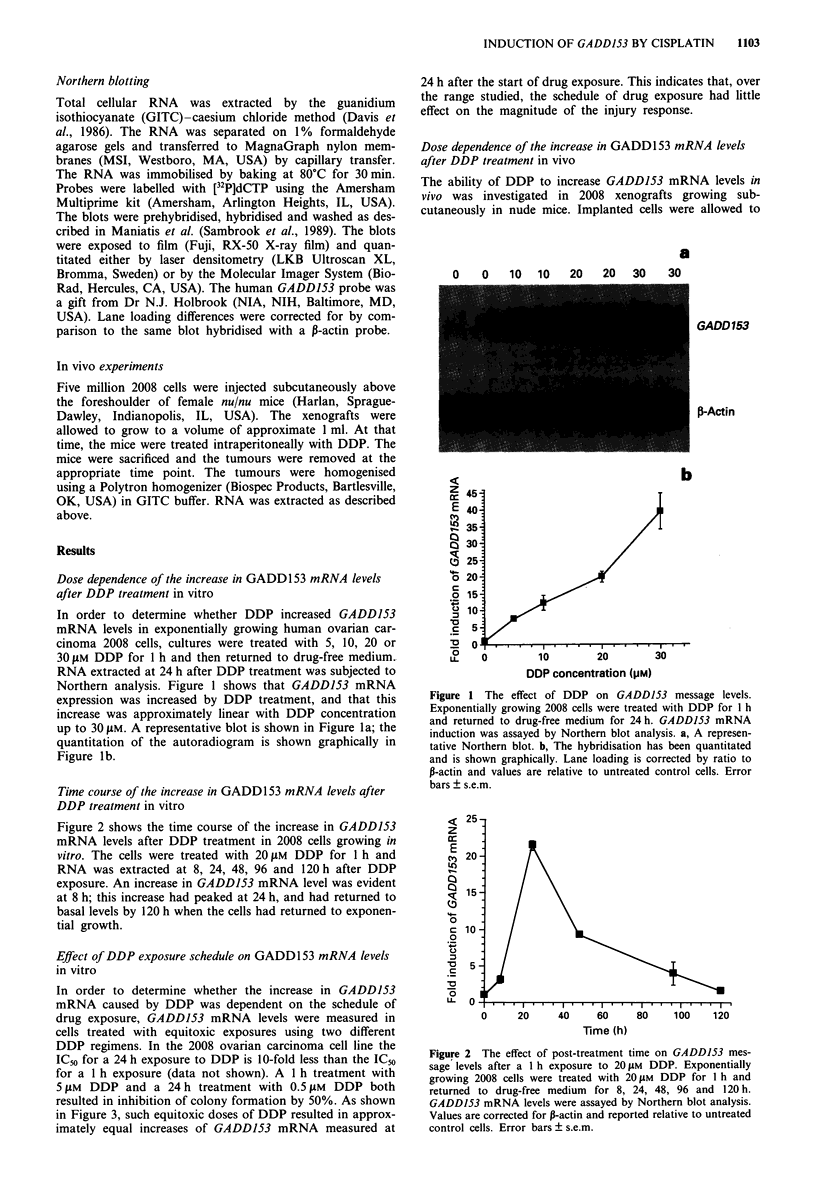

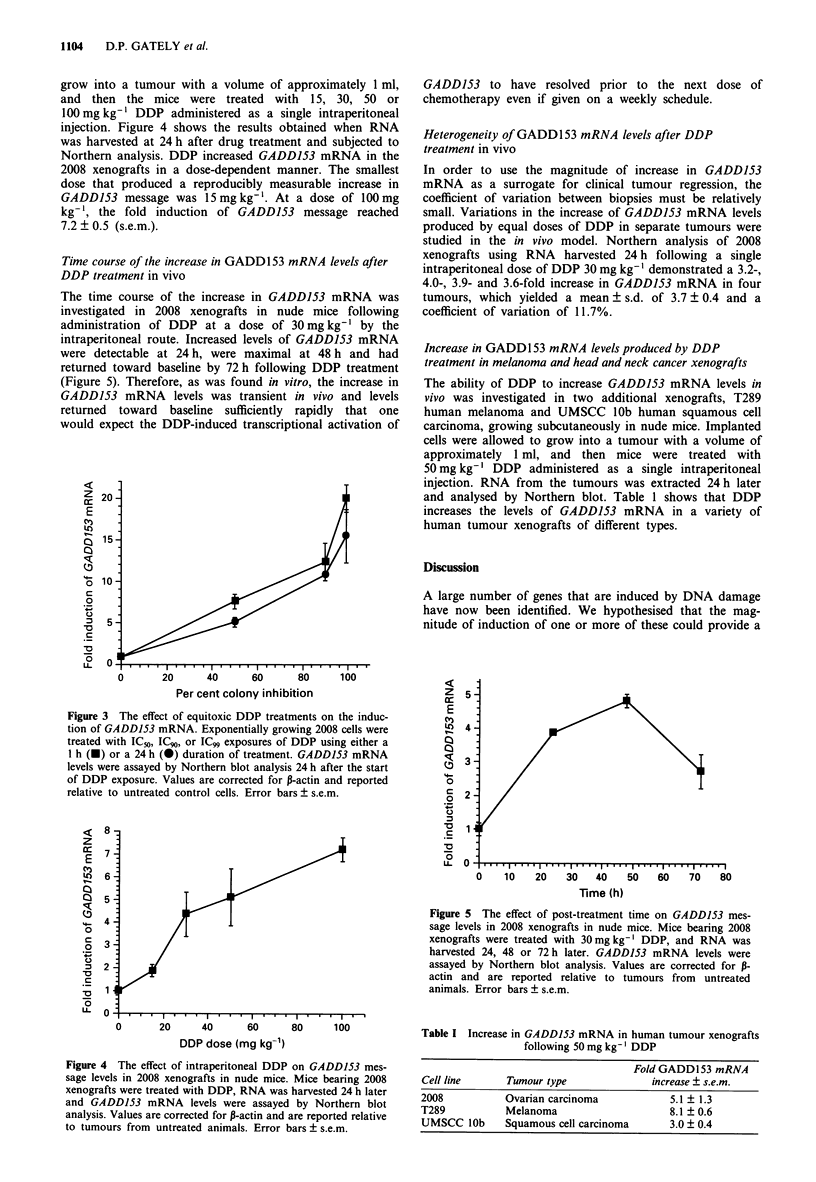

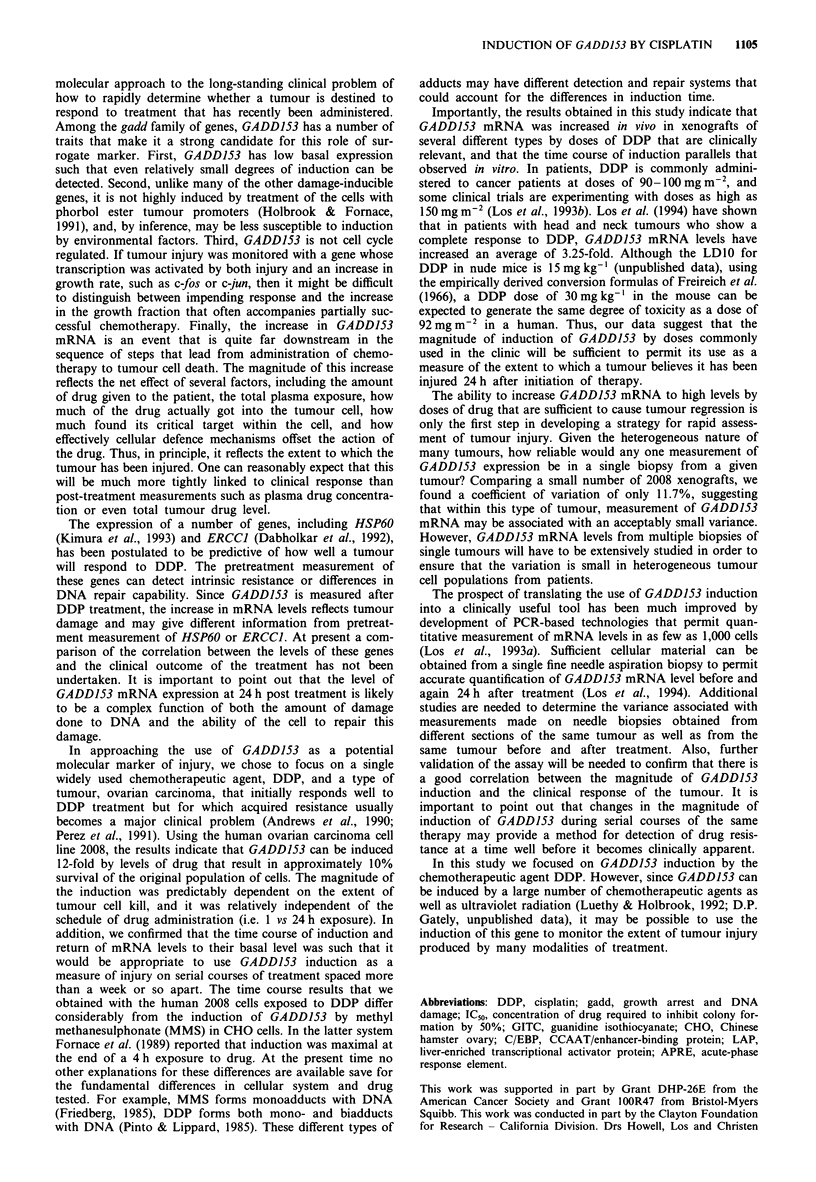

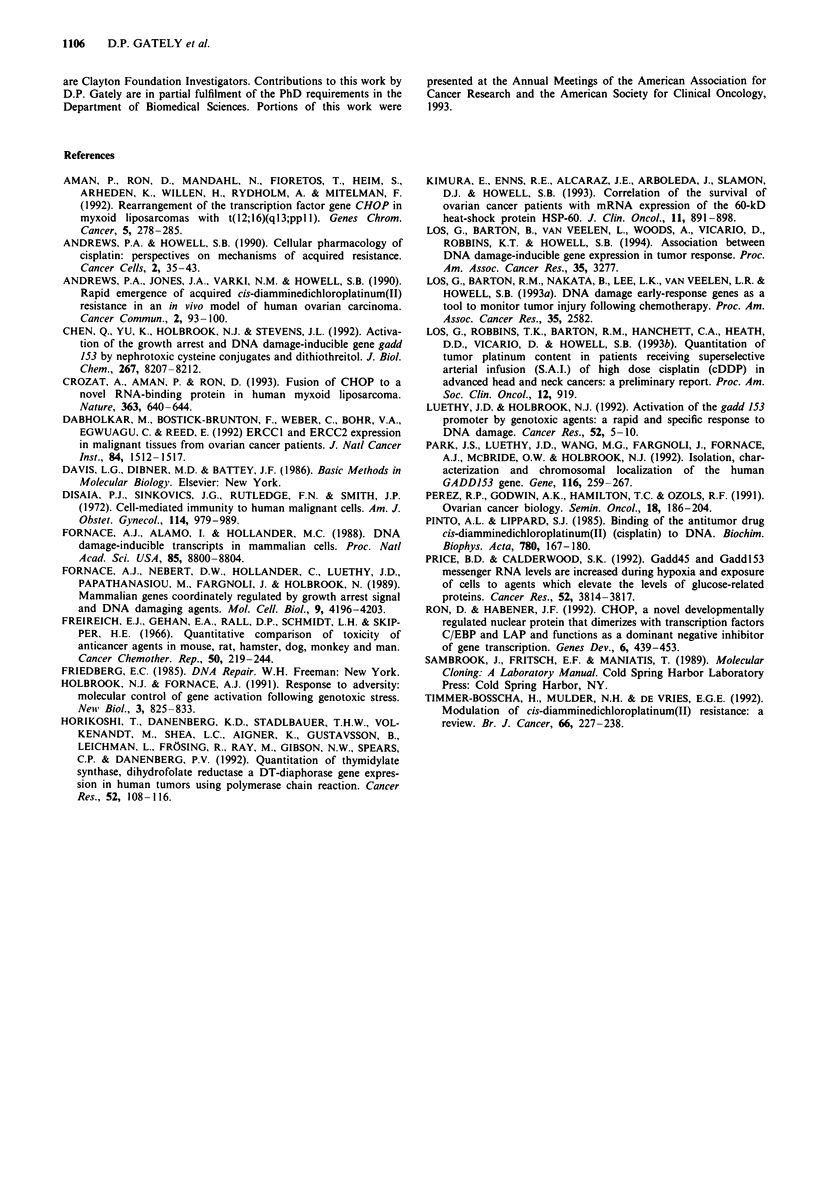


## References

[OCR_00653] Aman P., Ron D., Mandahl N., Fioretos T., Heim S., Arheden K., Willén H., Rydholm A., Mitelman F. (1992). Rearrangement of the transcription factor gene CHOP in myxoid liposarcomas with t(12;16)(q13;p11).. Genes Chromosomes Cancer.

[OCR_00660] Andrews P. A., Howell S. B. (1990). Cellular pharmacology of cisplatin: perspectives on mechanisms of acquired resistance.. Cancer Cells.

[OCR_00665] Andrews P. A., Jones J. A., Varki N. M., Howell S. B. (1990). Rapid emergence of acquired cis-diamminedichloroplatinum(II) resistance in an in vivo model of human ovarian carcinoma.. Cancer Commun.

[OCR_00671] Chen Q., Yu K., Holbrook N. J., Stevens J. L. (1992). Activation of the growth arrest and DNA damage-inducible gene gadd 153 by nephrotoxic cysteine conjugates and dithiothreitol.. J Biol Chem.

[OCR_00677] Crozat A., Aman P., Mandahl N., Ron D. (1993). Fusion of CHOP to a novel RNA-binding protein in human myxoid liposarcoma.. Nature.

[OCR_00682] Dabholkar M., Bostick-Bruton F., Weber C., Bohr V. A., Egwuagu C., Reed E. (1992). ERCC1 and ERCC2 expression in malignant tissues from ovarian cancer patients.. J Natl Cancer Inst.

[OCR_00692] DiSaia P. J., Sinkovics J. G., Rutledge F. N., Smith J. P. (1972). Cell-mediated immunity to human malignant cells. A brief review and further studies with two gynecologic tumors.. Am J Obstet Gynecol.

[OCR_00697] Fornace A. J., Alamo I., Hollander M. C. (1988). DNA damage-inducible transcripts in mammalian cells.. Proc Natl Acad Sci U S A.

[OCR_00702] Fornace A. J., Nebert D. W., Hollander M. C., Luethy J. D., Papathanasiou M., Fargnoli J., Holbrook N. J. (1989). Mammalian genes coordinately regulated by growth arrest signals and DNA-damaging agents.. Mol Cell Biol.

[OCR_00710] Freireich E. J., Gehan E. A., Rall D. P., Schmidt L. H., Skipper H. E. (1966). Quantitative comparison of toxicity of anticancer agents in mouse, rat, hamster, dog, monkey, and man.. Cancer Chemother Rep.

[OCR_00715] Holbrook N. J., Fornace A. J. (1991). Response to adversity: molecular control of gene activation following genotoxic stress.. New Biol.

[OCR_00722] Horikoshi T., Danenberg K. D., Stadlbauer T. H., Volkenandt M., Shea L. C., Aigner K., Gustavsson B., Leichman L., Frösing R., Ray M. (1992). Quantitation of thymidylate synthase, dihydrofolate reductase, and DT-diaphorase gene expression in human tumors using the polymerase chain reaction.. Cancer Res.

[OCR_00729] Kimura E., Enns R. E., Alcaraz J. E., Arboleda J., Slamon D. J., Howell S. B. (1993). Correlation of the survival of ovarian cancer patients with mRNA expression of the 60-kD heat-shock protein HSP-60.. J Clin Oncol.

[OCR_00755] Luethy J. D., Holbrook N. J. (1992). Activation of the gadd153 promoter by genotoxic agents: a rapid and specific response to DNA damage.. Cancer Res.

[OCR_00760] Park J. S., Luethy J. D., Wang M. G., Fargnoli J., Fornace A. J., McBride O. W., Holbrook N. J. (1992). Isolation, characterization and chromosomal localization of the human GADD153 gene.. Gene.

[OCR_00766] Perez R. P., Godwin A. K., Hamilton T. C., Ozols R. F. (1991). Ovarian cancer biology.. Semin Oncol.

[OCR_00770] Pinto A. L., Lippard S. J. (1985). Binding of the antitumor drug cis-diamminedichloroplatinum(II) (cisplatin) to DNA.. Biochim Biophys Acta.

[OCR_00775] Price B. D., Calderwood S. K. (1992). Gadd45 and Gadd153 messenger RNA levels are increased during hypoxia and after exposure of cells to agents which elevate the levels of the glucose-regulated proteins.. Cancer Res.

[OCR_00781] Ron D., Habener J. F. (1992). CHOP, a novel developmentally regulated nuclear protein that dimerizes with transcription factors C/EBP and LAP and functions as a dominant-negative inhibitor of gene transcription.. Genes Dev.

[OCR_00792] Timmer-Bosscha H., Mulder N. H., de Vries E. G. (1992). Modulation of cis-diamminedichloroplatinum(II) resistance: a review.. Br J Cancer.

